# Magnitude of low birthweight in malaria endemic settings of Nanoro, rural Burkina Faso: a secondary data analysis

**DOI:** 10.1038/s41598-021-00881-8

**Published:** 2021-10-29

**Authors:** Moussa Lingani, Serge H. Zango, Innocent Valéa, Daniel Valia, Maïmouna Sanou, Sékou O. Samandoulougou, Annie Robert, Halidou Tinto, Michèle Dramaix, Philippe Donnen

**Affiliations:** 1 École de Santé Publique, Université Libre de Bruxelles, Route de Lennik 808, CP594, 1070 Bruxelles, Belgique; 2 Epidemiology and Biostatistics Research Division, Institut de Recherche Expérimentale et Clinique, Université Catholique de Louvain, Brussels, Clos Chapelle-aux-Champs 30, B1.30.13, 1200 Brussels, Belgique; 3 Institut de Recherche en Sciences de la Santé/Direction Régionale du Centre Ouest (IRSS/DRCO), BP 218, 11 Nanoro, Burkina Faso; 4 Evaluation Platform on Obesity Prevention, Quebec Heart and Lung Institute Research Center, Quebec City, QC G1V 4G5 Canada

**Keywords:** Epidemiology, Medical research, Risk factors

## Abstract

Low birthweight (LBW) is a worldwide problem that particularly affects developing countries. However, limited information is available on its magnitude in rural area of Burkina Faso. This study aimed to estimate the prevalence of low birthweight and to identify its associated factors in Nanoro health district. A secondary analysis of data collected during a cross-sectional survey was conducted to assess the prevalence of low birthweight in Nanoro health and demographic surveillance system area (HDSS). Maternal characteristics extracted from antenatal care books or by interview, completed by malaria diagnosis were examined through a multi-level logistic regression to estimate odd-ratios of association with low birthweight. Significance level was set at 5%. Of the 291 neonates examined, the prevalence of low birthweight was 12%. After adjustment for socio-demographic, obstetric and malaria prevention variables, being primigravid (OR = 8.84, [95% CI: 3.72–21.01]), or multigravid with history of stillbirth (OR = 5.03, [95% CI: 1.54–16.40]), as well as the lack of long-lasting insecticide treated bed net use by the mother the night preceding the admission for delivery (OR = 2.5, [95% CI: 1.1–5.9]) were significantly associated with neonate low birthweight. The number of antenatal visits however did not confer any direct benefit on birthweight status within this study area. The prevalence of low birthweight was high in the study area and represents an important public health problem in Burkina Faso. In light of these results, a redefinition of the content of the antenatal care package is needed.

## Introduction

Despite halving the global neonatal mortality rate from 36.6 to 18.0 per 1000 live births between 1990 and 2017^1^, an estimated 2.5 million neonates died in the world in 2018, mainly among low birthweight neonates (LBW)^2^. Indeed, low birthweight—a birthweight below 2500 g—is an important risk factor for neonatal deaths^3^, which particularly affects developing countries where malaria transmission is endemic^4^. Sub-Saharan Africa is particularly affected due to the high risk of maternal infections during pregnancy^5,6^. Malaria, an example of these infections, causes up to 20% of all low birthweights in this region and 6% of neonatal deaths^7^. Thus, potentially, 100,000 infant lives could be saved each year if adequate malaria prevention measures were implemented. In Burkina Faso, malaria is the primary reason for seeking health care, and pregnant women are particularly affected.

To reduce malaria-related low birthweights in Sub-Saharan Africa, the World health organization (WHO) recommends a set of measures for pregnant women during antenatal care visits, which include the intermittent preventive treatment of malaria using sulfadoxine-pyrimethamine (IPTp-SP)^8^. IPTp-SP consists of monthly administration of sulfadoxine-pyrimethamine (SP) in pregnant women from the second trimester until delivery^8^. The strategy is effective in reducing malaria incidence and its consequences^9^. Burkina Faso adopted the strategy in 2005, with a minimum of three doses of sulfadoxine-pyrimethamine administered under the supervision of a healthcare worker^10^.

Although low birthweight adverse effects on neonatal survival have been well established^3^, little information is available on its prevalence in developing countries such as Burkina Faso. Indeed, at the start of the IPTp-SP policy in Burkina Faso, the prevalence of low birthweight was 15.8%^11^. Five years later, an evaluation reported a prevalence of 13.4%^12^. Since then, no other evaluation was conducted despite the numerous concerns raised by the low coverage of IPTp-SP strategy^13^ and the spread of *Plasmodium* *falciparum* resistance to sulfadoxine-pyrimethamine throughout the African continent^14^. Thus, a re-evaluation of the prevalence of adverse birth outcomes is necessary to timely seek alternative approaches. Therefore, this study aimed to assess the prevalence of low birthweight in rural Burkina Faso ten years after the IPTp-SP policy was adopted.

## Methods

### Study settings

The study was conducted in all eight peripheral health centers of the Nanoro health and demographic surveillance system (HDSS) area^15^. Nanoro is situated in the centre-west region of Burkina Faso at about 90 km from Ouagadougou, the capital city, and has a population of 63,000 inhabitants^15^. Malaria transmission is holo-endemic, with peak transmission overlapping with the rainy season (June-November). The commonest malaria vectors are *Anopheles gambiae*, *A. funestus, A. arabiensis* and *P. falciparum* is the predominant malaria parasite. Malaria is the main reason for visiting health centres with a case-fatality rate between 5 and 30%^16^.

### Study design

We conducted a secondary analysis of data collected among pregnant women attending health centers for delivery in the Nanoro HDSS area from September 2013 to April 2014. The primary study of this analysis was an ancillary topic annexed to a main project (NCT01703884) entitled ‘ANC & Malaria Diagnostic in Pregnancy” and aimed to improve the quality of antenatal care and diagnostic services for malaria in pregnancy^17^. All eight peripheral health centers of the HDSS were included. The study site’s map is described elsewhere^15^. For each enrolled pregnant woman, their babies were assessed within 24 h of delivery. Demographic, gyneco-obstetric, and relevant medical history data were collected from antenatal care books or face-to-face interviews with the mother during both high and low malaria transmission seasons.

### Study participants

Pregnant women aged 15–45 years, attending the health center for delivery with a gestational age of at least 37 weeks, living in the Nanoro HDSS area, and that provided written informed consent were eligible for inclusion. Those who delivered non-singleton babies, not willing to participate, or whose neonate was transferred before documentation of birthweight were not included. Of the 323 women included in the primary study, 9.9% (32/323) were excluded (4 non-singleton pregnancies, 6 stillbirths, 3 neonate deaths, 2 very ill newborns that were referred before clinical examination, 5 missing birthweights, 3 concurrent participations to other study and 9 preterm deliveries) and 291 (90.1%) were included.

### Sample size

At the time of policy change, term LBW proportion was 15.8% of all live births^11^. We hypothesized that the new policy would reduce the low birthweight proportion to 10% after ten-year of widespread implementation. The Cochran formula n = Z^2^ *p*(1-p)/i^2^ was used to calculate the sample size where p = 10% is the expected proportion, i = 3.5%, represents the margin of error, Z the z-score that corresponds to the 95% confidence interval (1.96), n is the minimal sample size. The minimum required sample size was 282. This was a secondary analysis of database collected on 291 participants.

### Data collection procedures and variables collected

Data were extracted from antenatal care books and by face-to-face interviews. Maternal age, educational level, occupation, and sulfadoxine-pyrimethamine uptake were collected from the antenatal care books. The use of insecticide-treated net (ITN) the night before visiting the health facility for delivery or any other variables not available in the antenatal care book were obtained by interview of the mother. Gestational age was estimated using the knowledge of the last menstrual period (LMP), or the Ballard score whenever LMP was unknown. Neonate examination was conducted within 24 h of delivery either in the health facility or at home by trained midwives. Birthweight was measured using a calibrated electronic scale with 10 g resolution and a precision less than 10 g (seca gmbh & co. kg, Germany). In addition, a malaria test using peripheral blood was performed by rapid diagnostic tests SD-Bioline Malaria Antigen *Pf®* test strips (sensitivity 99.7% and specificity of 99.5%). Table 1 presents the list, definitions, and grouping of variables. The outcome variable was birthweight at term categorized as normal birthweight (≥ 2500 g) and low birthweight (< 2500 g)^18,19^.

**Table 1:** List of exploratory variables.

No	Variable	Definition	Categories
1	Age	Mother age (years)	15–19 ; 20–34 ; and 35–42
2	Education	Highest level of education	None, primary or more
3	Occupation	Mother’s occupation during the last 12 months	Unemployed, employed/self employed
4	Gravidity	Number of pregnancies	Primigravidae (1); paucigravidae (2–4); multigravidae (≥ 5)
5	Stillbirth	Maternal history of stillbirth	Yes/no
6	Miscarriage	Maternal history of miscarriage	Yes/no
7	Kids	Number of living kids at the time of the survey	1; 2, 3, 4, ≥ 5
8	ITN use	Use of long lasting ITN the previous night	Yes/no
9	IPTp-SP	Number of IPTp—SP doses received	1; 2; ≥ 3
10	ANC	Number of ANC visits performed	< 4; ≥ 4
11	Malaria	Positive malaria rapid diagnostic test results	No/yes
13	Birthweight	Weight of neonate at birth in grams	Low (< 2500); normal (≥ 2500)

*ANC* antenatal care, *ITN* insecticide treated bed net, *IPTp-SP* intermittent preventive treatment of malaria with sulfadoxine-pyrimethamine.

### Data processing and analysis

Data were collected on a paper-based questionnaire by trained midwives and double-entered onto Redcap (Research—Electronic Data Capture) data collection tool, then imported into Stata version 15 (StataCorp. 2017, TX, USA) for cleaning and analysis. Categorical variables were summarized on frequency tables. Mean or median with respective measures of spread, including standard deviations and quartiles, were used to summarize numerical variables. Odds ratios (OR) and 95% confidence intervals (95% CI) were calculated according to each maternal factor using univariate logistic regression. Adjusted odds ratios (aOR) and 95% CI were derived by a backward multivariate logistic regression analysis of variables with statistically significant p-values at the univariate analysis, except for malaria. The final model retained variables which p-values were statistically significant. Variable age was not included due to its strong correlation with gravidity. A p-value < 0.05 was considered statistically significant.

### Consent to publication

All data were anonymized before publication, thus consent for publication was not applicable.

### Ethical considerations

Ethical clearance for this study was obtained from the National Ethics Committee of Burkina Faso. All data were anonymized before analysis. All participants or their authorized legally representatives provided a written informed consent prior to their participation. All methods were performed in accordance with the relevant guidelines and regulations.

## Results

### Study participants’ characteristics

Table 2 summarizes study participants’ background characteristics. Of the 291 mothers included, the mean age ± standard deviation was 26 ± 6 years, and most of them were unemployed (85.2%). Fourteen percent (14%) of women received at least three doses of sulfadoxine-pyrimethamine. Malaria infection was detected in one-third of the participants.

**Table 2:** Background characteristics of women included in the study (n = 291).

Characteristics	Items	n	Percentage
Age group (years)	< 20 20–34 ≥ 35	59 197 35	20.3 67.7 12.0
History of stillbirth	No Yes	260 31	89.3 10.7
History of miscarriage	No Yes	246 45	84.5 15.5
Gravidity	1 2–4 5 or more	66 145 80	22.7 49.8 27.5
Malaria test	Negative Positive	186 105	63.9 36.1
ITN use	Yes No	227 51	81.6 18.4
ANC	≤ 1 2 ≥ 3	15 51 225	5.2 17.5 77.3
IPTp-SP (doses)	≤ 1 2 ≥ 3	91 159 41	31.3 54.6 14.1
Education	None Primary or more	244 47	83.8 16.2
Occupation	Unemployed Employed/self employed	248 43	85.2 14.8

*ANC* antenatal care, *SP* sulfadoxine-pyrimethamine, *n* the absolute number of observations for variables.

### Low birthweight prevalence and associated factors

Of the 291 neonates, the mean birth weight (± standard deviation) was 2933 ± 390 g and 12.0% (35/291) live births were low birthweight. Table 3 presents the proportions of low birthweight and the maternal sociodemographic characteristics, obstetrical history as well as the univariate and adjusted odds ratios. Being primigravid or multigravid with an obstetrical history of stillbirth, and the lack of long-lasting insecticide-treated bed net use were significantly associated with low birthweight. However, the number of antenatal care visits performed did not show any link with the prevalence of low birthweight.

**Table 3:** Low birthweight prevalence and maternal factors by univariate and multivariate analysis, Burkina Faso (n = 286).

Characteristics	Total	LBW (%)	OR	95%CI	p. value	aOR	95%CI	p. value
**Age group (years)**								
< 20 20–34 ≥ 35	59 197 35	28.8 9.2 0.0	4.0 1.0 –	1.9–8.5 Ref –	< 0.001*	–	–	–
**Education**								
None Primary or more	244 47	11.5 14.9	0.7 1.0	0.3–1.8 Ref	0.509	–	–	–
**Occupation**								
Housewives Others	248 43	12.5 9.3	1.0 0.7	Ref 0.3–2.1	0.539	–	–	–
**Gravidity**								
Primigravid Multigravid with history of stillbirth Multipara	61 26 199	29.5 19.2 4.5	8.8 5.0 1.0	3.7–21.0 1.5–16.4 Ref	< 0.001*	8.83 5.0 Ref	3.7–21.0 1.5–16.4	< 0.001* < 0.001*
**History of miscarriage**								
No Yes	246 45	11.4 15.6	1.0 1.4	Ref 0.6–3.5	0.443	–	–	–
**Malaria**								
No Yes	186 105	10.8 14.3	1.0 1.4	Ref 0.7–2.8	0.378	–	–	–
**ITN use**								
Yes No	227 51	9.7 21.6	1.0 2.6	Ref 1.2–5.7	0.018*	Ref 2.5	1.1–5.9	0.04*
**IPTp-SP (doses)**								
≤ 1 2 ≥ 3	91 159 41	14.3 11.3 9.8	1.5 1.2 1.0	0.5–5.1 0.4–3.7 Ref	0.703	–	–	–

*SP* sulfadoxine-pyrimethamine, *CI* confidence interval, *ANC* antenatal care, *LBW* low birthweight, *Ref* reference group, *ITN* insecticide treated bed nets, *OR* odd ratios, *aOR* adjusted odd ratios.

*Statistically significant.

Multi-level logistic regression analysis included variable gravidity, the use of insecticide-treated bed nets the night before hospital admission for delivery, and malaria test results. Being primigravid (OR = 8.83, [95% CI: 3.71–21.01]), multigravid with an obstetrical history of stillbirth (OR = 5.03, [95% CI: 1.54–16.40]) and the lack of bed net use (OR = 2.5, [95% CI: 1.1–5.9]) were significantly associated with higher proportion of low birthweight among neonates.

## Discussion

Low birthweight prevalence was high in Nanoro given that those from preterm births were not included in this analysis. However, the report showed a substantial decrease (3.8 percentage points) from its level at the start of the IPTp-SP strategy^11^. Several factors, including the mother's socioeconomic status, obstetrical characteristics, and malaria prevention methods, influence low birthweight prevalence, and those factors differed from each setting. Indeed, lower low birthweight rates were observed in Ethiopia (10%)^20^ and in Nigeria (7%)^21^. However, these estimates were understated as the Nigerian’s study used data collected retrospectively from the routine surveillance system, which is not adapted to SSA as half of neonate birthweights are not recorded due to weaknesses of the reporting system^4^. Also, the study in Ethiopia was conducted in urban areas where women's economic level tends to be higher than those from the rural areas, and the impact of socioeconomic status on the prevalence of low birthweight is well established^22,23^.

In contrast, rates in excess were reported in South Africa (38.5%)^24^ and in a study conducted in Zimbabwe (16.7%)^25^. These could be related to the higher prevalence of human immuno-deficiency virus (HIV) infection^26^. Indeed, HIV is considered an important risk factor of LBW, although the mechanism is not yet fully elucidated^25,27^.

It is reported that maternal obstetrical history and events occurring during pregnancy impact the outcome of pregnancies, although those factors are not always identified^28^.

We found that the order of pregnancy was significantly associated with low birthweight and first pregnancy infants were at higher risk than others. The biological ground for first pregnancy neonates to be at higher risk of low birthweight may be related to the susceptibility of first pregnancy placentas to parasite-infected red blood cells sequestration, disrupting fetus nutrients supply^29^ and increasing risk of low birthweight, or because some physiologic changes are less efficient during first pregnancies thus increasing the risk of low birthweight^30^. Indeed, uteroplacental blood flow, responsible for delivering oxygen and nutrients to the fetus, is greater during subsequent pregnancies than in the first pregnancy^31,32^. Also, we have noted that mothers with multiple pregnancies were also at higher risk if there was an obstetrical history of stillbirth. Chen *et a*l. in 2018 and Rozi et al. in 2016 made such observations in their respective studies^33,34^, while Ahrens et al. went further to notice that the risk was highest after a history of three stillbirths^35^. Particular attention should be paid to pregnant women with such background characteristics during their antenatal care visits.

As expected, the lack of long-lasting insecticide-treated bed net usage was associated with an increased risk of low birthweight^36^. Bed net usage has long been recommended in areas with sustained malaria transmission as an effective tool to prevent malaria-related adverse birth outcomes, including low birthweight^37^. Although bed net is freely provided to pregnant women during the first antenatal care visit in Burkina Faso, a high proportion of women (up to 20%) were not covered, and actions toward improving bed net usage are required.

The number of antenatal care visits performed and the intermittent preventive treatment of malaria in pregnancy did not directly influence the prevalence of low birthweight in this study. Although the limited sample size did not allow an accurate evaluation of all low birthweight factors, *Plasmodium falciparum* resistance to sulfadoxine pyrimethamine reported in many parts of sub-Saharan Africa and Burkina Faso could be a limiting factor of the efficacy of IPTp-SP strategy^38–41^. Indeed, the high number of triple mutants, and the presence of quintuple mutants (triple *dhfr* and double *dhps* mutations) reported in Burkina Faso may have decreased the efficacy of sulfadoxine-pyrimethamine on *P. falciparum* in the study settings, and thus the effects of the IPTp-SP strategy on low birthweight^42^.

Contrary to what is reported in the literature, malaria was not associated with low birthweight in the current study. The use of malaria rapid diagnosis test could have understated the actual extent of malaria infection in the study population and thus, underestimated the extent of malaria negative effects on the fetus^43^. Studies building on the most accurate diagnosis methods are needed in this study setting.

A number of limitations in this study are worth noting and taken into consideration. The study was conducted on term pregnancies excluding low birthweight from preterm deliveries, which could understate the problem's actual extent. It remains to bear in mind that gestational age was dated from parental knowledge of the last menstrual period (LMP) or the Ballard score, which could be prone to some errors. The use of malaria RDT for the diagnosis in peripheral blood sample could have understated the extent of malaria infection in the study population and thus undermine the strength of association with low birthweight. Although the analysis had limitations, it depicted the overall picture of the magnitude of low birthweight in rural Burkina Faso.

## Conclusion

Low birthweight prevalence was high in Nanoro and required more effective policies. Exploring novel approaches or improving current approaches is needed. But first, quality data building on larger sample size are necessary to update maternal risk factors of low birthweight.

## Data Availability

The dataset used and analyzed during the current study is available from the corresponding author.

## Abbreviations

SP: Sulfadoxine pyrimethamine
IPTp: Intermittent preventive treatment of malaria in pregnancy
LBW: Low birthweight
ANC: Antenatal care
OR: Odds ratio
aOR: Adjusted odds ratio
CI: Confident interval
SDG: Sustainable development goals
NMR: Neonatal mortality rate
HDSS: Health and demographic surveillance system
HIV: Human immunodeficiency virus
WHO: World health organization
LMP: Last menstrual period

## References

[1] Hug L, Alexander M, You D, Alkema L (2019). National, regional, and global levels and trends in neonatal mortality between 1990 and 2017, with scenario-based projections to 2030: A systematic analysis. Lancet Glob. Health.

[2] *WHO-Newborns: Reducing Mortality 2019*. https://www.who.int/news-room/fact-sheets/detail/newborns-reducing-mortality. Accessed 30 Oct 2020.

[3] Katz J, Lee A, Lawn J, Cousens S, Blecowe H, Ezzati M (2013). Mortality risk in preterm and small-for-gestational-age infants in low-income and middle-income countries: A pooled country analysis. Lancet.

[4] *UNICEF-Low Birthweight*. 2019. https://data.unicef.org/topic/nutrition/low-birthweight/. Accessed 23 May 2020.

[5] Lee AC, Katz J, Blencowe H, Cousens S, Kozuki N, Vogel JP (2013). National and regional estimates of term and preterm babies born small for gestational age in 138 low-income and middle-income countries in 2010. Lancet Glob. Health.

[6] Lawn JE, Blencowe H, Shefali O, You D, Lee AC, Waiswa P (2014). Every newborn: Progress, priorities, and potential beyond survival. Lancet.

[7] Guyatt HL, Snow RW (2004). Impact of malaria during pregnancy on low birth weight in Sub-Saharan Africa. Clin. Microbiol. Rev..

[8] *WHO | Intermittent Preventive Treatment in Pregnancy (IPTp)*. 2019. https://www.who.int/malaria/areas/preventive_therapies/pregnancy/en/. Accessed 4 May 2020.

[9] Kassoum K, Garner P, Maria A (2015). Intermittent preventive therapy for malaria during pregnancy using 2 vs 3 or more doses of sulfadoxine-pyrimethamine and risk of low birth weight in Africa: Systematic review and meta-analysis. JAMA.

[10] Gansané A, Nébié I, Soulama I, Tiono A, Diarra A, Konaté AT (2009). Change of antimalarial first-line treatment in Burkina Faso in 2005. Bull Soc. Pathol. Exot..

[11] Kabore P, Donnen P, Dramaix-wilmet M (2007). Obstetrical risk factors for lowbirth-weight in a rural Sahelian area. Sante Publ. (Paris)..

[12] He Z, Bishwajit G, Yaya S, Cheng Z, Zou D, Zhou Y (2018). Prevalence of low birth weight and its association with maternal body weight status in selected countries in Africa: A cross-sectional study. BMJ Open.

[13] Yaya S, Uthman OA, Amouzou A, Bishwajit G (2018). Use of intermittent preventive treatment among pregnant women in sub-Saharan Africa: Evidence from malaria indicator surveys. Trop. Med. Infect. Dis..

[14] van Eijk AM, Larsen DA, Kayentao K, Koshy G, Slaughter DEC, Roper C (2019). Effect of *Plasmodium falciparum* sulfadoxine-pyrimethamine resistance on the effectiveness of intermittent preventive therapy for malaria in pregnancy in Africa: A systematic review and meta-analysis. Lancet Infect. Dis..

[15] Derra K, Rouamba E, Kazienga A, Ouedraogo S, Tahita MC, Sorgho H (2012). Profile: Nanoro health and demographic surveillance system. Int. J. Epidemiol..

[16] *WHO-Burkina Faso African Region*. 2019. https://www.who.int/malaria/publications/country-profiles/profile_bfa_en.pdf?ua=1. Accessed 30 Nov 2020.

[17] *ANC & Malaria Diagnostic in Pregnancy-Full Text View-ClinicalTrials.gov*. https://clinicaltrials.gov/ct2/show/NCT01703884. Accessed 18 Sep 2021.

[18] Rosenberg RE, Ahmed ASMNU, Ahmed S, Saha SK, Chowdhury MAKA, Black RE (2009). Determining gestational age in a low-resource setting validity of last menstrual period. J. Health Popul. Nutr..

[19] Cutland CL, Lackritz EM, Mallett-Moore T, Bardají A, Chandrasekaran R, Lahariya C (2017). Low birth weight: Case definition & guidelines for data collection, analysis, and presentation of maternal immunization safety data. Vaccine..

[20] Gebregzabiherher Y, Haftu A, Weldemariam S, Gebrehiwet H (2017). The prevalence and risk factors for low birth weight among term newborns in Adwa general hospital, northern Ethiopia. Obstet. Gynecol. Int..

[21] Dahlui M, Azahar N, Oche OM, Aziz NA (2016). Risk factors for low birth weight in Nigeria: Evidence from the 2013 Nigeria demographic and health survey. Glob. Health Action..

[22] Kaur S, Ng CM, Badon SE, Jalil RA, Maykanathan D, Yim HS (2019). Risk factors for low birth weight among rural and urban Malaysian women. BMC Public Health.

[23] Rezende Chrisman J, Mattos IE, Koifman RJ, Koifman S, Moraes Mello Boccolini P, Meyer A (2016). Prevalence of very low birthweight, malformation, and low Apgar score among newborns in Brazil according to maternal urban or rural residence at birth. J. Obstet. Gynaecol. Res..

[24] Tshotetsi L, Dzikiti L, Hajison P, Feresu S (2019). Maternal factors contributing to low birth weight deliveries in Tshwane district, South Africa. PLoS ONE.

[25] Feresu SA, Harlow SD, Woelk GB (2015). Risk factors for low birthweight in Zimbabwean women: A secondary data analysis. PLoS ONE.

[26] Eaton JW, Rehle TM, Jooste S, Nkambule R, Kim AA, Mahy M (2014). Recent HIV prevalence trends among pregnant women and all women in sub-Saharan Africa. AIDS.

[27] Hoque ME, Towbola OA, Mashamba TJ, Monokoane T (2014). Comparison of adverse pregnancy outcome between teenage and adult women at a tertiary hospital in South Africa. Biomed. Res..

[28] Scrimshaw, S.C., & Backes, E.P. *Committee on Assessing Health Outcomes by Birth Settings A Consensus Study Report*. 2020. 10.17226/25636. Accessed 11 Oct 2020.

[29] Duffy PE (2007). *Plasmodium* in the placenta: Parasites, parity, protection, prevention and possibly preeclampsia. Parasitology.

[30] Soma-Pillay P, Nelson-Piercy C, Tolppanen H, Mebazaa A (2016). Physiological changes in pregnancy. Cardiovasc. J. Afr..

[31] Clapp JF, Capeless E (1997). Cardiovascular function before, during, and after the first and subsequent pregnancies. Am. J. Cardiol..

[32] Prefumo F, Bhide A, Sairam S, Penna L, Hollis B, Thilaganathan B (2004). Effect of parity on second-trimester uterine artery Doppler flow velocity and waveforms. Ultrasound Obstet. Gynecol..

[33] Rozi S, Butt ZA, Zahid N, Wasim S, Shafique K (2016). Association of tobacco use and other determinants with pregnancy outcomes: a multicentre hospital-based case-control study in Karachi, Pakistan. BMJ Open.

[34] Chen S, Yang Y, Qu Y, Zou Y, Zhu H, Yang H (2018). Both maternal and paternal risk factors for term singleton low birthweight infants in rural Chinese population: A population-based, retrospective cohort study. Sci. Rep..

[35] Ahrens KA, Rossen LM, Branum AM (2016). Pregnancy loss history at first parity and selected adverse pregnancy outcomes. Ann. Epidemiol..

[36] Pryce J, Richardson M, Lengeler C (2018). Insecticide-treated nets for preventing malaria. Cochrane Database Syst. Rev..

[37] Gamble C, Ekwaru JP, ter Kuile FO (2009). Insectide-treated nets for preventing malaria in pregnancy. Cochrane Database Syst. Rev..

[38] Mandoko PN, Rouvier F, Kakina LM, Mbongi DM, Latour C, Likwela JL (2018). Prevalence of *P falciparum* parasites resistant to sulfadoxine/pyrimethamine in the democratic republic of the congo: Emergence of highly resistant *PfdHFR/PfdHps* alleles. J. Antimicrob. Chemother..

[39] Minja DTR, Schmiegelow C, Mmbando B, Boström S, Oesterholt M, Magistrado P (2013). *P falciparum* mutant haplotype infection during pregnancy associated with reduced birthweight, Tanzania. Emerg. Infect. Dis..

[40] Mbonye AK, Birungi J, Yanow SK, Shokoples S, Malamba S, Alifrangis M (2015). Prevalence of *P falciparum* resistance markers to sulfadoxine-pyrimethamine among pregnant women receiving intermittent preventive treatment for malaria in Uganda. Antimicrob. Agents Chemother..

[41] Xu C, Sun H, Wei Q, Li J, Xiao T, Kong X (2019). Mutation profile of pfdhfr and pfdhps in *P. falciparum* among returned Chinese migrant workers from Africa. Antimicrob. Agents Chemother..

[42] Ruizendaal E, Tahita MC, Geskus RB, Versteeg I, Scott S, D’Alessandro U (2017). Increase in the prevalence of mutations associated with sulfadoxine-pyrimethamine resistance in *Plasmodium falciparum* isolates collected from early to late pregnancy in Nanoro, Burkina Faso. Malar. J..

[43] Fried M, Muehlenbachs A, Duffy PE (2012). Diagnosing malaria in pregnancy: An update. Expert Rev. Anti Infect. Ther..

